# Structural and functional heterogeneity in phloem loading and transport

**DOI:** 10.3389/fpls.2013.00244

**Published:** 2013-07-05

**Authors:** Thomas L. Slewinski, Cankui Zhang, Robert Turgeon

**Affiliations:** Department of Plant Biology, Cornell UniversityIthaca, NY, USA

**Keywords:** phloem loading, heterogeneity, sieve elements, companion cells

## Abstract

The phloem is often regarded as a relatively straightforward transport system composed of loading (collection), long-distance (transport), and unloading (release) zones. While this simple view is necessary and useful in many contexts, it belies the reality, which is that the phloem is inherently complex. At least three types of sieve element–companion cell complexes are found in minor veins of leaves. Individual species may have more than one type, indicating that they employ multiple loading strategies, even in the same vein. Gene expression data in particular point to heterogeneity in sieve element–companion cell complexes of minor veins, perhaps in all flowering plants. Phloem heterogeneity in the transport phloem is also evident in many species based on anatomical, biochemical and gene expression data. In this regard, members of the Cucurbitaceae are especially complex and interesting. We conclude that a hidden world of specialized phloem function awaits discovery.

## INTRODUCTION

Early anatomists noted many differences in phloem structure within and between species. These differences include, but are by no means limited to, sieve tube dimensions and content, sieve plate structure, wall composition and, at the organization level, the association of different types of parenchyma cells, the presence or absence of internal phloem, and differences in structure and longevity of protophloem, metaphloem, and secondary phloem. In addition, the intricate sieve tube systems in members of the Cucurbitaceae are a continuing source of fascination. In this paper we focus primarily on recent studies of heterogeneity in the loading phloem of leaves and also review recent attempts to unravel structure/function relationships in the transport phloem of the cucurbits.

## PHLOEM LOADING AND MINOR VEINS

### PHLOEM LOADING MECHANISMS

Three phloem loading mechanisms are known and there is evidence (see below) that more than one mechanism can be, and often is, used by a single species (van Bel, 1993; Turgeon, 2010a; Aoki et al., 2012). Each mechanism has associated with it a distinctive type of companion cell.

In apoplastic loading, sucrose produced in mesophyll cells enters the cell wall space (apoplast) and is taken up into the minor vein phloem by transporters. This is a thermodynamically active process that uses the proton gradient as an energy source. Several authors have comprehensively reviewed sucrose transporters (Sauer, 2007; Braun and Slewinski, 2009; Kuhn and Grof, 2010; Slewinski and Braun, 2010). The companion cells associated with apoplastic loading are usually unspecialized in appearance, except for those with cell wall ingrowths. The cytoplasm may be dense, and these unspecialized cells are often referred to as “ordinary” companion cells (Grusak et al., 1996). These minor vein companion cells are symplastically connected to adjacent cells, in addition to their sieve element, but the plasmodesmata are not numerous. This makes sense since a highly porous interface at this location would create a futile pump-leak cycle. In some species the walls of companion cells engaged in loading have prominent ingrowths that facilitate uptake from the apoplast. When wall ingrowths are present the cells are called “transfer cells” (Pate and Gunning, 1969). The presence of companion cells specialized as transfer cells seems, according to present evidence, to be a clear indication that loading occurs from the cell wall space.

Polymer trapping is another loading strategy with a very distinct companion cell type. These companion cells, known for historical reasons as “intermediary cells,” are larger than most ordinary companion cells or phloem parenchyma cells. They have dense cytoplasm, many small vacuoles, and extremely abundant plasmodesmata that connect them to adjacent bundle sheath cells (Turgeon et al., 1993). The plasmodesmata are highly branched, more so on the intermediary cell side than on the bundle sheath side. Polymer trapping occurs when sucrose diffuses into the intermediary cells through these cytoplasmic connections and is then converted to raffinose and stachyose (Zhang and Turgeon, 2009).

In passive loading, sucrose and other transport compounds such as sugar alcohols simply diffuse into the phloem from the mesophyll without conversion to other compounds (Rennie and Turgeon, 2009). These companion cells have abundant, but symmetrically branched, plasmodesmata.

### ACTIVE VERSUS PASSIVE LOADING

Phloem loading via the apoplast or by polymer trapping elevates the concentration of sugar in the companion cells to much higher levels than in the mesophyll and is therefore thermodynamically active. As the name implies, passive loading is thermodynamically downhill at the companion cell interface; transport compounds follow their concentration gradient into the companion cell. Therefore the sucrose, and in some species the combined sucrose and sugar alcohol concentration, in leaves of passive loaders is considerably higher than that of most active loaders (Rennie and Turgeon, 2009). Nonetheless, the phloem pressure osmotically generated by passive loaders is relatively low (Turgeon, 2010b).

This raises an interesting point concerning the role of phloem pressure. It has been generally assumed that active loading evolved to increase solute concentration, and therefore hydrostatic pressure, in the phloem to motivate long-distance transport by the Münch mechanism. However, passive loading is almost exclusively associated with trees, where the transport distances are longest. This suggests that the pressure difference between source and sink tissues needed to drive Münch pressure flow may be relatively low (Turgeon, 2010b) and that phloem pressure in many species of smaller stature exceeds that need. The true phloem pressure differential required for long-distance transport is a matter of continuing discussion (Mullendore et al., 2010; Jensen et al., 2012).

### WHY THREE LOADING MECHANISMS?

Evolutionary theory tells us that loading mechanisms must confer advantages to the plants that adopt them. The obvious advantage to symplastic loading is that material is able to move from the surrounding cells into the phloem along an open-access channel. Did symplastic loading evolve specifically to load sucrose in this manner? This seems unlikely given that sucrose is efficiently loaded along the apoplastic route in so many plants. More likely is the possibility that other beneficial molecular species slip into the phloem through the plasmodesmata and thereby avoid what may be a hostile or difficult-to-traverse route through the cell walls and membranes. At present these putative molecular species remain unknown; their identification is potentially an exciting and unexplored aspect of phloem physiology. Defense compounds or signal macromolecules capable of directed transport through plasmodesmata are candidates.

As noted above, the advantage to active loading has seemed obvious for a long time: it generates the pressure needed to drive transport over long distances. However, the capacity of many large plants to operate effectively with less pressure suggests that other explanations are needed (Turgeon, 2010b). What other purposes might phloem pressure fill? There are many possibilities and all of them could play a part. An obvious and often-discussed role for pressure is that its release forces the coagulated contents of the sieve tubes against the sieve plates as part of the wound sealing mechanism.

Another way of looking at phloem pressure is that adaptive advantage derives from the high sugar content of the sieve tubes. Elevated sugar concentrations present a severe osmotic challenge to potential phloem feeders. Also, the theoretical optimal concentration of sugar that maximizes mass transfer of carbohydrate while minimizing the resultant impedance to flow is close to that found in the phloem (Jensen et al., 2013).

Yet another possibility is that the benefit derives not from high phloem sugar but from low non-structural carbohydrate (NSC) content in the rest of the leaf (Turgeon, 2010a). Reducing NSC content to its minimum in the lamina (or anywhere else in the plant) increases growth potential by freeing up fuel needed for rapid growth. Indeed, herbaceous plants, as a rule, maintain the lowest possible levels of leaf NSC consistent with nighttime needs (Fondy and Geiger, 1982; Smith and Stitt, 2007). Given such low sucrose content in mesophyll cells, some form of active loading is needed to bring the sugar content and pressure in the phloem up to levels needed to drive transport effectively. Since rapid growth is such a strong determinant of success in herbaceous plants (Lambers and Poorter, 1992), one would expect passive loading, with its requirement for high foliar sucrose content, to be non-adaptive. Indeed, passive loading is rare in herbs.

If the rapid growth argument is valid for herbs, why not for trees? Why do so many woody plants use the passive mechanism, which requires elevated laminar sucrose content? One reason appears to be that trees must maintain high foliar solute levels to offset low whole-plant hydraulic conductance (Fu et al., 2011). In effect, they elevate solute concentrations in the leaf to facilitate water transport in the xylem. Given the need for high laminar sugar concentrations, trees cannot take advantage of the low NSC-strategy used by herbaceous plants to potentiate growth. As a corollary, they have no need to use energy to increase sugar content and pressure in the phloem.

Do these strategies confer ecological advantages? Early correlations of loading types with climate indicated that symplastic loading is uncommon in cold-climate plants (Gamalei, 1989). In these analyses polymer trapping and passive loading species were conflated into a single group. When they are separated it is clear that there is a weak correlation of polymer trapping with tropical climates, for unknown reasons (Davidson et al., 2011). More evident is the virtual absence of passive loading species in the arctic (Davidson et al., 2011). This can be explained by the fact that virtually all passive loading species are trees, which are absent in the arctic for reasons unassociated with phloem loading or transport.

The adoption of different loading strategies also has consequences in terms of adaptation to the light environment (Amiard et al., 2005). In effect, the different structural components are variables or tools that can be manipulated to adjust to changing conditions. For example, in plants with wall invaginations in the companion cells (transfer cells), the ingrowths become more extensive and convoluted in high light, which increases export capacity (Wimmers and Turgeon, 1991; Offler et al., 2003; Patrick, 2013). Polymer trap plants appear to be constrained by the inability to produce more plasmodesmata and as a result mature leaves are unable to acclimate as readily when the plants are transferred from low to high light (Amiard et al., 2005). The plants acclimate eventually by growing new leaves with higher vein density, which increases plasmodesmatal frequency per unit area of leaf surface.

## LOADING HETEROGENEITY IN SINGLE SPECIES

As the apoplastic loading model was being developed, it was assumed that the mechanism was ubiquitous. Then, when other loading strategies were found, they were thought to be unique to particular species or families. Thus, it became common to identify the different mechanisms with specific plants. Solanaceous species were termed “apoplastic loaders,” cucurbits were “polymer trappers” and later, trees such as willow and poplar were termed “passive loaders.” However, it is becoming increasingly clear that some, and perhaps all, plants use more than one mechanism. Therefore it is more correct to use the loading terms to describe the mechanisms themselves, rather than the particular species in which they are found, even if that mechanism is dominant.

Early hints of heterogeneity in loading mechanisms were evident in the 1990s when tests of the apoplastic loading mechanism were conducted in transgenic solanaceous plants by expressing invertase in the apoplast (von Schaewen et al., 1990; Dickinson et al., 1991) or downregulating the sucrose transporter (Riesmeier et al., 1994). These treatments severely inhibited phloem loading, photoassimilate export and growth. However, in many cases the plants that were most profoundly affected were nonetheless able to grow, if slowly, at least in low light. This result has since been replicated in other species. For example, although *Arabidopsis* is considered to be an apoplastic loader (Wippel and Sauer, 2012), mutants with no sucrose (SUC2) transporter activity can be grown to reproductive maturity on soil (Srivastava et al., 2009). Also, polymer trap plants in which raffinose and stachyose synthesis is essentially eliminated are weak, but in low light they survive and grow (McCaskill and Turgeon, 2007).

These results suggest that at least some plants harbor a redundant phloem loading mechanism(s), a conclusion supported by early structural evidence. In a key paper, van Bel et al. (1992) noted that both intermediary cells and transfer cells are present in the minor veins of *Acanthus mollis*, strongly suggesting a “mixed loading” strategy of polymer trapping and apoplastic loading. Indeed, it seems likely that all polymer trap plants use both mechanisms simultaneously. In the cucurbits, intermediary cells are found on the abaxial side of the minor veins, while a single ordinary companion cell occupies the adaxial position (Turgeon et al., 1975; Schmitz et al., 1987). In progressively larger vein orders, ordinary companion cells increase in proportion to the number of intermediary cells which consistently occupy the flanks of the vein. This is true in other families also, as illustrated in *Coleus blumei* (Fisher and Eschrich, 1985). In still larger veins, which function more in long-distance transport than phloem loading, the intermediary cells disappear altogether.

How much photoassimilate is loaded by the ordinary companion cells in polymer trap plants? An estimate can be derived by analyzing the sugar profile of the transport stream since ordinary companion cells load sucrose without conversion to raffinose family oligosaccharides (RFOs). In most RFO species the amount of radiolabeled stachyose greatly exceeds the amount of radiolabeled sucrose downstream of the leaf blade after it has been labeled with ^14^CO_2_ (Turgeon et al., 1993), suggesting that polymer trapping is the dominant mechanism. However, these results are difficult to interpret without knowledge of the specific activities of the labeled chemical species. Direct measurements of sugars from aphid stylets inserted into the phloem of the polymer trapping plant *Alonsoa meridionalis* indicate that 21% of the sugar is sucrose and 77% is raffinose and stachyose combined (Voitsekhovskaja et al., 2006). These results confirm that most loading occurs in the intermediary cells. Some of the sucrose could as well since it is present in intermediary cells as a substrate for RFO synthesis and there should be no impediment for diffusion of sucrose from intermediary cells into the sieve tubes. Nonetheless, this amount of sucrose in aphid stylet sap suggests that ordinary companion cells contribute meaningfully to the transport stream.

Another way to test this hypothesis is to downregulate the sucrose transporter and thereby interfere with the function of ordinary companion cells. Downregulating SUT1 in *Verbascum phoeniceum *did not result in typical symptoms of transport inhibition such as starch accumulation, inhibition of photosynthesis or leaf chlorosis (Zhang and Turgeon, 2009). A small amount of soluble carbohydrate accumulated in the leaves of the most severely affected transgenics, indicating that apoplastic loading may play a role in transport, but if so it appears to be relatively small.

**FIGURE 1:**
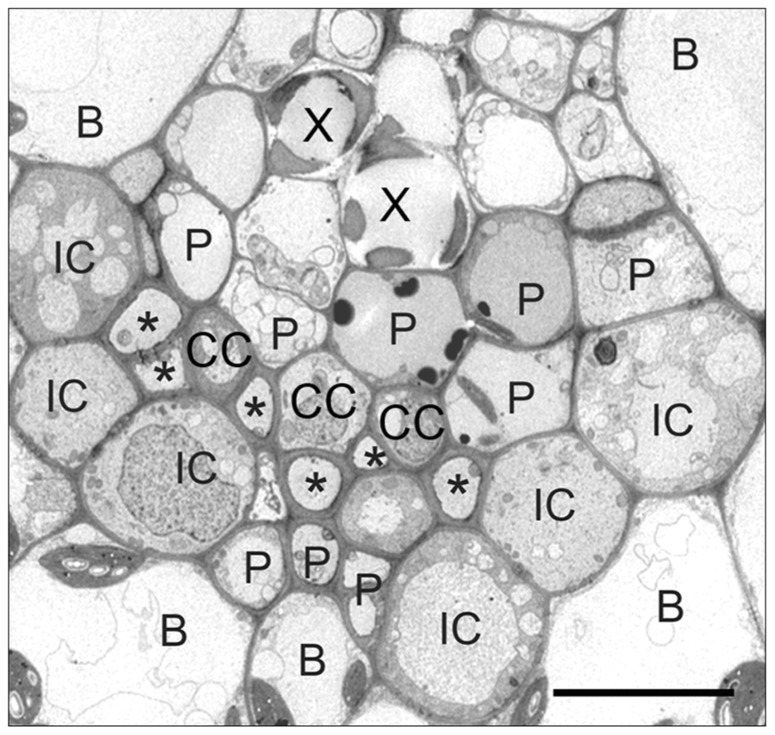
** Transmission electron micrograph of an *Amborella trichopoda *vein.** (X) xylem, (P) parenchyma cell, (CC) ordinary companion cell, (IC) intermediary cell, (B) bundle sheath, (*) sieve element. Note the ring of ICs that line the abaxial side of the vein, with companion cells on the inside of the vein. Scale bar = 2 μm (from Turgeon and Medville, 2011; reprinted with permission).

As noted above, in some polymer trap species, especially in the Scrophulariaceae and related families (van Bel et al., 1992; Turgeon et al., 1993), transfer cells replace the ordinary companion cells in the minor veins and can be even larger than the intermediary cells. Thus the proportional representation of polymer trapping and apoplastic loading is species specific. At one end of the spectrum, intermediary cells dominate and the plants translocate stachyose with little sucrose. These species include, but are not limited to, the cucurbits, *Catalpa speciosa* (Turgeon and Medville, 2004), and many members of the Scrophulariaceae (Turgeon et al., 1993). In species with transfer cells in the minor veins in addition to intermediary cells, including *Asarina* spp.**(Turgeon et al., 1993; Voitsekhovskaja et al., 2006), and several other members of the Scrophulariaceae (Turgeon et al., 1993), there is less [^14^C]stachyose and more [^14^C]sucrose in the transport stream following exposure of the leaf to ^14^CO_2_. *Amborella trichopoda*, the only member of the Amborellaceae and sister to all other extant angiosperms, is another interesting case. In this shrub, found only in New Caledonia, the smallest veins are composed of a cluster of ordinary companion cells, enveloped on the abaxial side by a string of intermediary cells (**Figure 1**; Turgeon and Medville, 2011). This species transports almost equal amounts of sucrose and stachyose.

**FIGURE 2:**
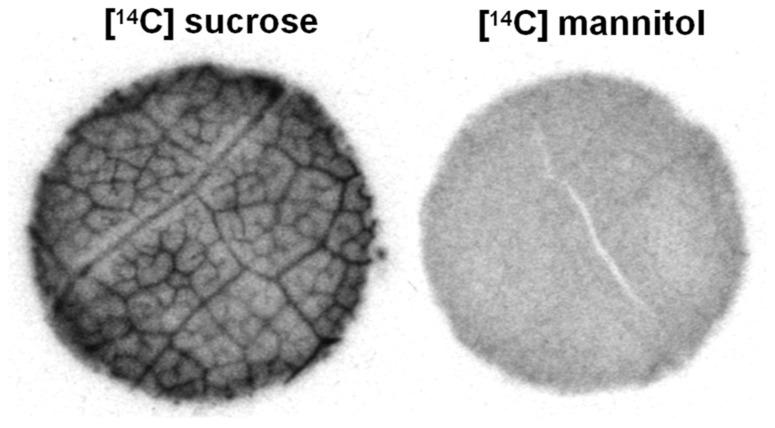
** Autoradiograph of *Asarina scandens* leaf discs following uptake of [^14^C]sucrose or [^14^C]mannitol.** Radiolabel from [^14^C]sucrose, but not [^14^C]mannitol, accumulates in the veins (figure modified from Rennie and Turgeon, 2009; reprinted with permission).

*Asarina* spp. and *Fraxinus* spp. are especially interesting since, on the basis of several lines of structural and physiological evidence, these plants use all three loading strategies. They transport sucrose, RFOs, and mannitol (Zimmermann and Ziegler, 1975; Turgeon et al., 1993). As discussed above, it is reasonable to assume that at least some of the sucrose loads via the apoplastic pathway. This is especially true in the case of *Asarina,* which has large transfer cells in the minor veins and in which sucrose is the primary transport sugar (Turgeon et al., 1993; Voitsekhovskaja et al., 2006). In both species, sucrose also enters the intermediary cells through the plasmodesmata because they synthesize and transport RFOs. The mannitol appears to enter the intermediary cells through plasmodesmata, as does sucrose. The reason for suspecting this pathway for mannitol is that, when leaf discs are provided with exogenous [^14^C]mannitol, the radiolabel does not accumulate in the minor veins as would be expected if it loads actively by transporters (**Figure 2**). In contrast, when this experiment is conducted with species that transport sugar alcohols but do not have companion cells with symplastic connections between the mesophyll and phloem, such as *Plantago major* (Reidel et al., 2009; Fu et al., 2011) and *Apium graveolens* (celery; Rennie and Turgeon, 2009), the radiolabel accumulates in the veins, in the same way as sucrose. Therefore, in species with intermediary cells, sugar alcohol presumably loads symplastically and passively and is carried away in the phloem along the pressure gradient formed by active RFO synthesis in the intermediary cells.

**FIGURE 3:**
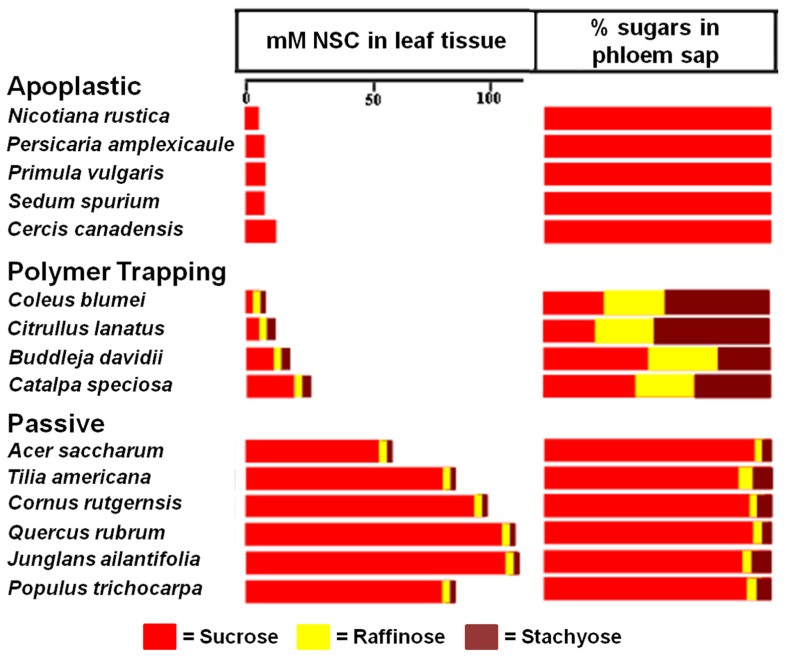
** Transport sugars in the leaf tissue and phloem of species exhibiting different strategies of phloem loading.** Non-structural carbohydrate (NSC) concentrations (from Rennie and Turgeon, 2009) are compared with the percentages of the same sugars in phloem exudate (from Zimmermann and Ziegler, 1975). Note the absence of raffinose and stachyose in the plants, including tree species, that load from the apoplast, whereas trees that load passively have detectable levels of these sugars. The proportion of raffinose and stachyose in the phloem sap of polymer trapping plants is higher in the phloem than in the leaf because they are synthesized in the phloem.

When Zimmermann and Ziegler (1975) conducted a large survey of transport sugars, primarily in trees, they found RFOs in the phloem sap of many species. As expected, these species include polymer trappers such as *Catalpa* and *Fraxinus*. However, a number of trees that are likely passive loaders, based on structural grounds, were also found to contain small but significant amounts of RFOs in the phloem, primarily raffinose (**Figure 3**). Significantly, tree species in the Zimmermann and Ziegler survey that do not transport detectable amounts of RFOs are in families associated with apoplastic loading (**Figure 3**) lending credence to the hypothesis that RFOs play a role in symplastic phloem loading even in “passive” loading species where they are only present in small concentrations.

Interestingly, when the first step in RFO synthesis, production of galactinol, was upregulated in tobacco, a species that predominantly uses apoplastic loading, significant amounts of raffinose were detected in the phloem (Ayre et al., 2003). Raffinose synthesis is downstream of galactinol synthesis in the RFO pathway. This suggests that although the RFOs are normally below the threshold of detection in the phloem sap in tobacco, and perhaps other plants, the underlying mechanism for RFO synthesis is present in some form within the phloem tissue (Turgeon et al., 2001; Ayre et al., 2003).

Other herbaceous plants that are considered apoplastic loaders, including *Arabidopsis*, also transport small amounts of raffinose in addition to sucrose (Haritatos et al., 2000b).* Arabidopsis* has a diverse population of cells in the vasculature, including phloem parenchyma cells with wall ingrowths (Haritatos et al., 2000b; Maeda et al., 2006). At present the site of synthesis of raffinose in *Arabidopsis* and in the trees that transport small amounts of RFOs is not known, and the function of these sugars in phloem loading and transport is obscure.

Why maintain a heterogeneous phloem loading and transport system? Different phloem loading strategies may confer different advantages under various environmental conditions. Thus, it is likely that multiple phloem loading mechanisms allows plants to rapidly adapt to biotic and abiotic stresses. In melon plants, infection with cucumber mosaic virus increases the proportion of sucrose to raffinose and stachyose by upregulating sucrose loading (Shalitin and Wolf, 2000; Shalitin et al., 2002) while the activities of the enzymes in the raffinose and stachyose synthesis pathway remain unaffected (Gil et al., 2011).

## PHLOEM HETEROGENEITY IN MONOCOTS

Monocot leaves are very different from most dicot leaf blades (Nardmann et al., 2004). According to the phyllode hypothesis, at an early point in their history monocots suffered a dramatic reduction of the upper lamina and what we now refer to as a “leaf blade” is an extrapolation of the petiole (Arber, 1918) or “lower leaf zone” (Kaplan, 1973), with the lower leaf blade now restricted to the tip of the leaf (Arber, 1918). The lower leaf zone or petiolar origin of the present day blade would explain why monocot leaves have parallel, rather than reticulate venation (Arber, 1918; Kaplan, 1973). Phloem loading occurs in the minor and intermediate veins; whereas transport out of the leaf occurs in the large lateral veins. Transfer of the photoassimilate from the collection phloem to the transport phloem within the large veins occurs via the transverse veins that connect adjacent parallel veins (Kuo et al., 1972). Interestingly, some of the features of minor veins that are common in dicot leaf blades appear to be missing in monocots (Evert et al., 1978). No known monocots have intermediary cells or use the polymer trapping mechanism. Although, similar to the patterns in eudicots, a few monocots have been found to contain transfer cells within the phloem region of the leaf blade (Pate and Gunning, 1969; Wilson et al., 1985; Lalonde et al., 2001).

Continuing this line of reasoning, the loss or drastic reduction of the upper leaf blade may have also altered minor vein-specific programs of phloem loading, specifically the enzymes required for polymer trapping and sucrose transporters involved in apoplastic loading. Perhaps this is why the monocots that have been analyzed, primarily grasses, do not employ transporters in the Group 2 clade, those that drive apoplastic loading in eudicots (Braun and Slewinski, 2009). Because Group 2 transporters are missing in the monocots, if they load from the apoplast they must use transporters from other clades, most likely those that mediate sucrose retrieval in the transport phloem. The ability of alternative sucrose transporters to load the phloem was recently demonstrated in *Arabidopsis*. Phloem loading was restored in the* Atsuc2 *mutant background when *AtSUT1* was expressed under the control of the *AtSUC2* promoter (Wippel and Sauer, 2012). In monocots, specifically grasses, only the SUT1 transporter, a member of the monocot specific Group 1 clade, has been shown to function in phloem loading (Slewinski et al., 2009). The orthologs of this protein in sugar cane (ShSUT1) and rice (OsSUT1) do not function in phloem loading and the transporters that carry out this function have not yet been identified (Braun and Slewinski, 2009).

**FIGURE 4:**
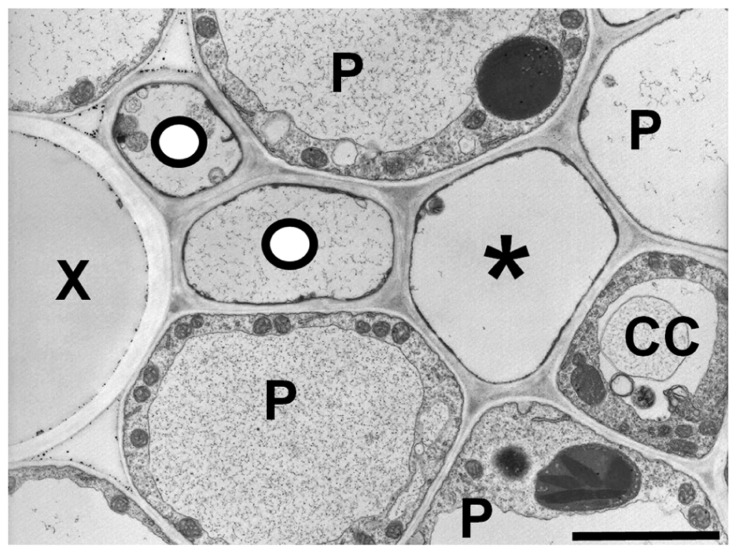
** Transmission electron micrograph of a maize minor vein showing thick- and thin-walled sieve elements.** Thick-walled sieve elements (O) are closely associated with the xylem (X). (P) parenchyma cell, (CC) companion cell, (*) thin-walled sieve element. Scale bar = 2.5 μm.

Grasses also appear to have a heterogeneous transport system (Evert et al., 1978), as most veins in the leaves have two types of sieve elements: thin- and thick-walled (**Figure 4**). The thin-walled sieve elements have ordinary companion cells (Evert et al., 1978). When the leaf is exposed to ^14^CO_2_, the thin-walled elements in the minor and intermediate veins become heavily labeled, suggesting they are the major conduits for photoassimilate export (Fritz et al., 1989). In contrast, thick-walled sieve elements lack companion cells, have abundant plasmodesmatal connections to the adjacent parenchyma cells (Evert et al., 1978). Additionally, the relative number of thick-walled sieve elements declines in the largest classes of veins as well as the transport phloem. The major role of thick-walled sieve elements may be in retrieval from the xylem (Botha et al., 2008), but they also become lightly labeled in the ^14^CO_2_ experiments (Fritz et al., 1989), and therefore may have a limited export role. This could help explain how maize plants that are homozygous for the *zmsut1* mutation are still able to survive and produce fertile spikelets in the tassels under certain environmental conditions (Slewinski et al., 2009). In barley, aphids preferentially feed on the thin-walled sieve tubes, suggesting there are different metabolite profiles between the two types of sieve tubes (Matsiliza and Botha, 2002). Mir1-CP, a defense cysteine protease accumulates in thick-walled, but not thin-walled sieve elements in maize (Lopez et al., 2007). There are many plasmodesmata between thick-walled sieve elements and vascular parenchyma cells and dye movement studies suggest a retrieval function from the xylem (Evert et al., 1978; Botha et al., 2008). However, the function of the thick-walled sieve elements in long-distance phloem transport, if any, remains unclear.

Is it possible that the thick-walled sieve elements in grasses share identity with both phloem and xylem? Analysis of the *Arabidopsis altered phloem development *(*apl*) mutants indicates that phloem and xylem overlap in their developmental programs (Bonke et al., 2003). APL is a MYB coil–coil type transcription factor that is essential for companion cell development and sieve element differentiation. Arabidopsis *apl *mutants fail to undergo the asymmetric cell division that gives rise to companion cells and they produce a phloem-to-xylem phenotype in that the mutant phloem strands (sieve elements), have xylem-like characteristics such as elaborate cell wall thinking that is usually found in the tracheary elements (Bonke et al., 2003). When *APL* is ectopically expressed throughout the vascular core, the protein represses xylem differentiation and induces phloem-like characteristics in the xylem tissue (Bonke et al., 2003; Carlsbecker and Helariutta, 2005). It has also been shown that immature xylem cells can transdifferentiate into phloem during bark regeneration in trees (Pang et al., 2008). Given the interplay between phloem and xylem differentiation (Carlsbecker and Helariutta, 2005; Miyashima et al., 2013), it is tempting to speculate that intermediates between both vascular identities could arise, leading to rapid diversity in both structure and physiology. Interestingly, the thick-walled sieve elements reside close to the xylem bundle in grasses (**Figure 4**). Thus the proposed mobile signals that lead to the differentiation of the phloem region of the bundle may not suffice to fully induce differentiation of the thick-walled precursor cells into thin-walled type phloem. Additionally, the thick-walled sieve elements in grasses appear to share many of the same characteristics as the modified phloem in the *apl* mutant. Both lack companion cells, have parenchyma cells similar to those usually associated with the xylem, and have thickened cell walls (Evert et al., 1978; Bonke et al., 2003; Botha et al., 2008). The walls of the thick-walled sieve elements appear to be lignified in wheat (*Triticum aestivum*; Kuo and O’Brien, 1974), but not in *Zea mays* (Walsh, 1974). As a test of this hypothesis it would be interesting to determine whether there are similar or shared gene expression patterns for thick-walled sieve elements and the xylem.

## HETEROGENEITY IN GENE EXPRESSION

In *Alonsoa*, a polymer trap plant, sucrose transporter mRNA (Voitsekhovskaja et al., 2006) and protein (Knop et al., 2004; Voitsekhovskaja et al., 2006) are present in the companion cells that mediate apoplastic loading, but not in the intermediary cells. Conversely, the stachyose synthase protein is found in the intermediary cells (Holthaus and Schmitz, 1991) and absent from the ordinary companion cells (Voitsekhovskaja et al., 2009).

These differences in gene expression occur in a specific type of plant – those that load by polymer trapping – in which heterogeneity is expected based on observable structural and physiological heterogeneity. More intriguing is evidence of heterogeneity in apparently uniform cell populations. For example, in *Plantago major*, an apoplastic loader, one subset of companion cells in the minor veins exhibits sucrose and sorbitol transporter activities, but another subset is missing the sorbitol transporters (Ramsperger-Gleixner et al., 2004).

**FIGURE 5:**
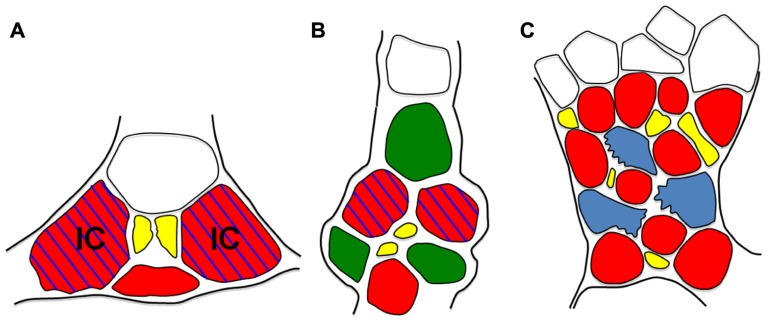
** Schematic diagrams of minor veins from*Verbascum * (A), Tobacco (B), and *Arabidopsis *(C).** Minor vein phloem. CCs are red, PP cells green, sieve elements are yellow, parenchyma cells are blue, xylem is white, IC = intermediary cell. Blue hatching indicates *pGAS::GUS* activity. Note that, except for the presence of PP cells in tobacco, cell arrangement and GUS staining patterns are the same in *Verbascum* and Tobacco. In *Arabidopsis*, parenchyma cells (blue) are also transfer cells. Cell wall ingrowths (jagged edges) localize on the phloem interface.

**FIGURE 6:**
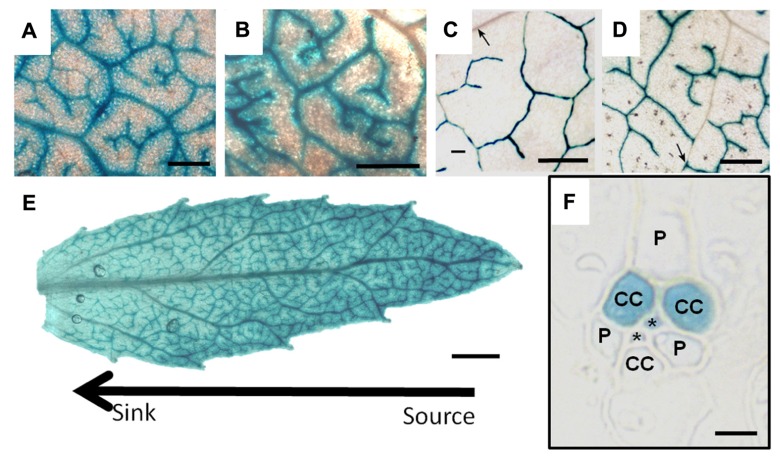
** GUS staining driven by the *galactinol synthase* promoter from *Cucumis melo* in leaves of (A) Poplar, (B) *Verbascum*, (C) *Arabidopsis*, and (D) Tobacco.** **(E)** is the staining pattern in a developing poplar leaf demonstrating that the tip (source) expresses the GUS gene more strongly than the base (sink). **(F)** is a transverse section of tobacco leaf tissue illustrating specific GUS staining in two of three companion cells (CC). (P) parenchyma cell, (*) sieve element. Scale bars = 400 μm in **(A–D)**; 1 mm in **(E)**; and 5 μm in **(F)** (**C**, **D**, and **F** are from Haritatos et al., 2000a. Reprinted with permission).

Another example, and one that suggests widespread, possibly ubiquitous, heterogeneity in minor vein phloem, comes from studies of the *galactinol synthase* (*GAS*) gene. GAS catalyzes the first step in stachyose production in polymer trap plants. The promoter (*pGAS*) is exquisitely localized to intermediary cells (Beebe and Turgeon, 1992; Haritatos et al., 2000b; McCaskill and Turgeon, 2007). But *pGAS* is also active in plants that do not load by polymer trapping. In tobacco, an unrelated species that produces no detectable galactinol or stachyose in minor veins, *pCmGAS* from melon is strongly active (**Figures 5B** and **6D,F**; Haritatos et al., 2000b). These results indicate that there is a conserved regulatory program in tobacco minor veins that activates *pCmGAS*. Interestingly, only two of the three companion cells activate *pCmGAS* in tobacco (**Figures 5B** and **6F**). Therefore, although the companion cells in the tobacco minor vein look identical, they are heterogeneous in their programming. Note that this is the same localization pattern as in melon and other polymer trap plants (**Figure 5A**). Remnants of this underlying RFO mechanism in the phloem may also explain why RFOs were found in the phloem sap of tobacco that ectopically produced galactinol in the leaves, as discussed earlier (Ayre et al., 2003).

Evidence for heterogeneity extends well beyond tobacco. *pCmGAS* is active in the minor veins of every species in which it has been tested (*Arabidopsis*, *Asarina*, poplar, tobacco and *Verbascum*; **Figure 6**). The purpose of the genetic program that activates *pGAS* is unknown, but its activity in all loading types suggests a program that has deep, conserved functions. Perhaps certain subsets of minor vein phloem cells synthesize different compounds related to defense or signaling. The activities of other phloem-specific promoters such as *rolc*, and those from the several viruses (Dutt et al., 2012) have not been tested for cell specificity in minor veins.

Vascular parenchyma cells also display heterogeneity. Phloem parenchyma cells in the minor veins of some species (Pate and Gunning, 1969), including *Arabidopsis *(Haritatos et al., 2000b), differentiate into transfer cells in a non-continuous pattern along the length of the phloem (Chinnappa et al., 2013) with wall ingrowths primarily at the interface with the sieve element–companion cell complex (**Figure 5C**). These ingrowths are more extensive in high light (Amiard et al., 2005; Adams et al., 2007), suggesting that they facilitate unloading into the apoplast as the initial step in phloem loading. Recently, a subset of the newly discovered SWEET transporters (Chen et al., 2012) has been shown to fulfill the function of efflux proteins that release sucrose into the apoplast within the phloem. However, analysis of *SWEET11* and *12* promoter activities show that these genes are only expressed in a subset of parenchyma cells (Chen et al., 2012), presumably transfer cells, again suggesting some degree of functional heterogeneity. Wall ingrowths in the phloem parenchyma cells of minor veins appear to have more than one function since deposition of wall material is also enhanced by exposure to cold (Maeda et al., 2006). The involvement of jasmonic acid in the signaling system also suggests a role in defense against pathogens and/or insects (Amiard et al., 2005; Adams et al., 2007). Thus, phloem parenchyma transfer cells may also have other functions that support phloem physiology apart from a direct role in carbohydrate loading.

## HETEROGENEITY IN THE TRANSPORT PATHWAY

As described in early anatomical studies, the phloem in shoots and roots of angiosperms is far from uniform (Esau, 1965). Variation is apparent in the arrangement of primary vascular bundles, the presence or absence of internal phloem, the presence or absence of vascular bundles in the pith, and production of secondary phloem, either by a single cambium or by successive cambial layers. This structural complexity undoubtedly underlies functional diversity, but aside from a few notable examples, this area of phloem physiology has not been extensively explored.

Some of the ultrastructural and physiological specialization found in the collection phloem of the leaf is reflected in more subtle forms within the transport phloem (Kempers et al., 1998). For example, plants that transport mostly sucrose maintain stronger membrane potentials and lower symplastic continuity in the transport phloem than plants with intermediary cells (Hafke et al., 2005). Perhaps the larger raffinose and stachyose molecules are less prone to leakage through the plasma membrane of the transport phloem than sucrose. If this is the case there would be less need for a strong membrane potential to drive retrieval back into the phloem, but it would also necessitate symplastic continuity to supply surrounding cells with sugar.

Another common example of heterogeneity in the transport pathway is the presence of phloem internal as well as external to the xylem (bicollateral phloem) in a number of families. In tomato, import into sink leaves occurs in the internal phloem while export occurs in the external phloem, and during the sink–source transition transport is bidirectional (Houngbossa and Bonnemain, 1985). The same pattern is seen in virus transport. For example, Cucumber mosaic virus travels in the external, rather than the internal phloem of *Tetragonia expansa* (Ohki, 2010) and tobacco mosaic virus is exported from *Nicotiana benthamiana* leaves in the external phloem and imported into sink leaves in the internal phloem (Cheng et al., 2000).

Biochemical specialization associated with the phloem is apparent in a few well-studied species. For example, in the Madagascar periwinkle (*Catharanthus roseus*), early steps in the synthesis of monoterpene indole alkaloids, secondary metabolites with therapeutic value, take place in parenchyma cells associated with the internal phloem (Geu-Flores et al., 2012; Simkin et al., 2013). Botha et al. (1977) also suggest that cardenolides are transported in the internal phloem.

The cucurbits are especially well known for the complexity of their phloem in petioles, stems, and fruit (Crafts, 1932). The plants have bicollateral vascular bundles composed of internal and external phloem (fascicular phloem), plus an extensive network of sieve tubes outside the bundles (extrafascicular phloem). The extrafascicular phloem of cucurbits consists of longitudinally oriented peripheral sieve tubes at the margins of the fascicular phloem and entocyclic sieve tubes just inside the sclerenchyma ring, connected by laterally oriented commissural sieve tubes. Extrafascicular phloem also includes the longitudinally oriented ectocyclic sieve tubes found outside the sclerenchyma ring (Zhang et al., 2012).

Recent studies have shown that the two phloem systems, fascicular and extrafascicular, differ in both composition and function. In pumpkin, the phloem proteins PP1 and PP2 and their mRNAs are found in all phloem types but are most abundant in the cortical bundles and the bundles associated with the extrafascicular sieve elements (Bostwick et al., 1992; Clark et al., 1997). However, recent compositional analysis on exudates and stylet samples in pumpkin indicate that PP1 and PP2 are present in the extrafascicular, but not in fascicular, phloem exudate (Walz et al., 2004; Gaupels et al., 2012). This suggests that although the *PP1* and *PP2* genes are transcribed and translated in both phloem types, the proteins do not exude from the fascicular phloem. Prominent proteins of about 32 and 60 kDa found in stylectomy samples from the fascicular phloem are not abundant in extrafascicular exudates (Gaupels et al., 2012). Also, the common sieve element occlusion (SEO/Common P) protein, separate from PP1 and PP2, involved in wound sealing of the phloem in pumpkin is present only in the fascicular phloem (Ernst et al., 2012).

Although researchers have recently clarified that the sap from the incised stem or petiole of pumpkin originates from the extrafascicular phloem (Zhang et al., 2010, 2012; Gaupels et al., 2012; Zimmermann et al., 2012), this discovery is being inaccurately generalized to all cucurbit species (Zhang et al., 2010; Atkins et al., 2011; Gaupels et al., 2012; Gaupels and Ghirardo, 2013). A recent study showed that the extrafascicular phloem origin of the sap is not a universal phenomena in the* Cucurbitaceae *(Zhang et al., 2012). In addition to pumpkin and cucumber, trials were also conducted on other species in the *Cucurbitaceae*. Zucchini (*Cucurbita pepo*) exuded primarily from the extrafascicular phloem in a pattern much like pumpkin, whereas watermelon (*Citrullus lanatus*), bitter apple (*Citrullus colocynthis*), luffa (*Luffa acutangula*), calabash (*Lagenaria siceraria*), and winter melon (*Benincasa hispida*) exuded primarily from the fascicular phloem, in a pattern much like cucumber. This study also showed that contamination from hexose, and presumably other compounds, is still a problem even in cucumber in which the sap mainly exudates from the fascicular phloem (Zhang et al., 2012). Although there is a higher stachyose/hexose ratio in cucumber versus pumpkin phloem sap, it is not entirely safe to conclude that there is less contamination in cucumber because stachyose is also a storage compound and could also be a contaminant. The extremely diluted characteristics of cucumber sap, similar with pumpkin sap, further suggests that most of the fluid comes from sources outside of phloem.

There is little doubt that fascicular phloem in cucurbits is the major translocation stream for carbohydrates, but what is the function of extrafascicular phloem? Following ^14^CO_2_ fixation in the lamina, radiolabel appears in the extrafascicular, as well as the fascicular phloem (Webb and Gorham, 1964), but this does not necessarily indicate that photoassimilate flows along this sieve tube network. It could be that there is exchange between the two systems and that the extrafascicular phloem functions more as a biochemical factory than as a distribution network (Turgeon and Oparka, 2010). Indeed, recent proteomic and metabolomic analyses of the extrafascicular phloem in cucurbits, which the authors refer to with justification as a type of laticifer, revealed many defense proteins and secondary compounds (Gaupels et al., 2012).

Metabolome analyses have also shown that the majority of small compounds detected by gas chromatography/mass spectrometry (GC/MS) in extrafascicular phloem exudates are either missing or at much lower concentrations in profiles of fascicular tissues (Zhang et al., 2010). Although PP1 and PP2 both exude from the extrafascicular phloem in pumpkin, only PP2 was identified from cucumber exudates. This further indicates a complicated origin of the exudates from these two species (Walz et al., 2004).

There are also differences in protein composition between the internal fascicular and external fascicular phloem sieve tubes (Zhang et al., 2010). After incision of cucumber stems, approximately twice as many droplets are observed, and 3.7 times as much exudate volume is collected, over the internal fascicular phloem compared to the external fascicular phloem, indicating a difference in sieve tube sealing (Zhang et al., 2012).

## HETEROGENEITY AND THE EVOLUTION OF DOMINANT PHLOEM LOADING TYPES

Phylogenetic analyses indicate that the evolution of loading types has a complex history (Gamalei, 1989, 1991; Turgeon et al., 2001). The presence of extensive plasmodesmatal connections between minor vein companion cells and surrounding cells appears to be ancestral in the angiosperms. This suggests that the first flowering plants used the passive loading strategy and that apoplastic loading and polymer trapping evolved later. However, this conclusion must be viewed with caution given the widespread occurrence of heterogeneity in minor veins, both in structure and function. It is possible that even the earliest angiosperms were capable of phloem loading by more than one mechanism and that what we consider the loading type of any plant, including basal species, may simply be a dominant form.

If minor vein phloem heterogeneity is as widespread as we suggest, then the emergence of a dominant form in one group or another in the present era likely occurs as an elaboration, rather than the *de novo* fabrication, of specific mechanisms. For example, polymer trapping has evolved independently several times as a recognizable trait (Turgeon et al., 2001), but other plants translocate small amounts of RFOs (Zimmermann and Ziegler, 1975). Therefore, the appearance of polymer trapping as a principal mechanism, as it is in many species of the Asteridae, could be accomplished by the upregulation of the RFO pathway, coupled to an increase in the number of existing plasmodesmata, and a slight modification of their internal structure. The evolution of apoplastic loading, from this perspective, occurs by the loss of plasmodesmata and the concurrent upregulation of sucrose transporters that are required in the transport phloem for retrieval of lost solute.

## CONCLUSION

The phloem is best characterized for its role in carbohydrate transport and partitioning but also has broader roles in the transport of minerals, defense compounds and signal macromolecules throughout the plant. Multiple mechanisms may be necessary to transport this diverse array of compounds and macromolecules from cell to cell. Previously, phloem heterogeneity was only considered an oddity or curiosity, occurring in select species. Yet the structural and physiological patterns seen in the more exaggerated examples appear to be conserved throughout vascular plants, albeit in more subtle forms.

Heterogeneity in phloem loading and transport appears to be more widespread than previously thought and many aspects of physiology and development that underpin the diversity within cells of the phloem tissues remain unknown. Understanding the complexity in this system will shed light on how plants partition resources, adapt and evolve to their environment and cope with biotic and abiotic stress.

## Conflict of Interest Statement

The authors declare that the research was conducted in the absence of any commercial or financial relationships that could be construed as a potential conflict of interest.
